# The Contractile Response to Oxytocin in Non-pregnant Rat Uteri Is Modified After the First Pregnancy

**DOI:** 10.1007/s43032-023-01163-6

**Published:** 2023-01-25

**Authors:** Maura Porta, Amber Boening, Jonathan Tiemann, Adam Zack, Arjun Patel, Korie Sondgeroth

**Affiliations:** 1 Department of Physiology, College of Graduate Studies, Midwestern University, Downers Grove, IL 60515 USA; 2 Master of Biomedical Sciences Program, Midwestern University, Downers Grove, IL 60515 USA; 3 Chicago College of Osteopathic Medicine, Midwestern University, Downers Grove, IL 60515 USA

**Keywords:** Pregnancy, Oxytocin, Oxytocin receptor, Uterus, Contractility, Calcium

## Abstract

During pregnancy, the uterus undergoes several modifications under the influence of hormonal and mechanical stimuli. We hypothesize that while most of these modifications are reverted during involution, some of the physiological properties of the uterus are permanently altered. To investigate this hypothesis, we conducted motility experiments to evaluate the contractility response of uterine tissue samples from non-pregnant virgin and proven breeder female rats to oxytocin (10^−10^ to 10^−5^ M). We found that the virgin tissue contracts more robustly than proven breeder tissue in the absence of oxytocin, yet with oxytocin, proven breeder samples displayed a significantly higher increase in activity. These results could depend on a more elevated expression of oxytocin receptor and/or on an alteration in the intracellular pathways affected by the activation of the oxytocin receptors. Here, we explored the impact of some structures involved in the management of intracellular calcium on the dose response to oxytocin recorded from virgin and proven breeder uterine strips. Specifically, we replicated the dose response experiments in low extracellular calcium (10 μM), in the presence of the intracellular calcium channel blocker ruthenium red (10 μM), and in the presence of the sarcoplasmic-endoplasmic reticulum calcium ATP-ase pump inhibitor, cyclopiazonic acid (10 μM). The results of these experiments suggest that also the expression of proteins that control intracellular calcium availability is affected by the experience of pregnancy. Molecular biology experiments will give us more detail on the magnitude of these expression changes.

## Introduction

Although it is well known that the complex array of hormonal and mechanical stimuli of pregnancy exerts profound impacts on the female physiology, our comprehension of the extent and permanency of these impacts is still incomplete. Epidemiologic studies have revealed a correlation between parity and an increase in the incidence of Alzheimer disease [1]. Gestational diabetes, a condition that is at least in part caused by hormones of pregnancy, such as human placental lactogen and prolactin, is a risk factor for development of diabetes type II later in life [2, 3]. Furthermore, although the high levels of estrogen and progesterone during pregnancy increase the short-term risk of breast cancer, in the long run, women who have been pregnant and have breastfed face a lower risk of developing breast cancer [4, 5].

In the uterus, pregnancy constitutes a protective factor against fibroids (leiomyomas), the most prevalent tumors in younger women [6]. In terms of uterine function, the duration of labor has been shown to be reduced in parous women [7]. In addition, the intensity of postpartum lower abdominal pains (commonly referred to as “after-pains”), very mild after the first delivery, is magnified after the second and ensuing parturitions. Postpartum aches are generated by powerful uterine contractions occurring as part of the involution process [8] and stimulated by oxytocin during lactation [9]. The characteristics of these contractions differ in the first and subsequent involution periods. During the involution period of first pregnancies, the uterine walls produce tonic contractions [10, 11]. Instead, during the involution periods of subsequent pregnancies, the uterus displays phasic and robust contraction. Possible hypotheses to explain this phenomenon include: (1) an increased release of oxytocin during lactation in multiparous women; (2) an increased ability of the multiparous uterus to respond to oxytocin; (3) a combination of these two effects; and (4) altered spontaneous contractility of the multiparous myometrium. There is evidence that oxytocin release is not affected by parity [12]. Others have documented that during the third trimester of pregnancy in first-time mothers, the plasma levels of oxytocin were higher than in multiparous women [7]. However, the current plasma oxytocin assessment methods are still unreliable [13], so a definitive statement on this point is difficult to make. Either way, a change in the myometrial ability to respond to oxytocin is likely, since the duration of labor is known to be reduced in multiparous women [14, 15].

On the other hand, parity is considered a risk factor for uterine atony, one of the most common causes of potentially fatal hemorrhages in parturition [16]. The exact etiology of this disturbance is unknown. However, the explanation for this dangerous complication might lie within epigenetic changes that affect intracellular calcium management, contractile proteins, or receptors for endogenous modulators such as oxytocin, prostaglandins, catecholamines, and sex hormones.

Dysmenorrhea, painful uterine cramping during the menstrual period, is estimated to affect between 15 and 75% of women (with peaks of 90% in women ≤ 25 years old), is inversely associated with age, use of oral contraceptives, and parity [17]. In particular, it has been observed that after the birth of the first child, the incidence and intensity of pain during menstruation is significantly reduced [18]. It has been hypothesized that this improvement could derive from a reduction in the ability of the endometrium to produce prostaglandins, the classical cause of primary dysmenorrhea [19–21]. According to another hypothesis, the drastic reduction in adrenergic fibers and uterine noradrenaline that occurs in preparation for labor could become a permanent feature, therefore attenuating dysmenorrhea after birth [18, 22].

A first step in gaining a clearer understanding of these phenomena is to investigate the effects of parity of uterine contractility in a carefully controlled system. In this study, we focused on the response of uteri from non-pregnant virgin (V) and proven breeder rats (PB) to oxytocin, one of the most used pharmacological tools to stimulate and evaluate uterine performance in vivo and in vitro.

Oxytocin is a nonapeptide synthesized by the magnocellular neurons of the supraoptic and paraventricular nuclei in the hypothalamus and secreted by the posterior pituitary. More recently, it has been found to be released by other tissues, such as the placenta, the corpus luteum, and the uterus itself [23]. Oxytocin exerts neuromodulator actions in the brain and hormone actions on various peripheral tissues, including the cardiovascular system, pancreas, liver, and bones [24]. In the uterus, oxytocin acts as a uterotonic and plays an important role during parturition.

Oxytocin receptors (OXTR) are part of the G protein-coupled receptor family and work via G_αq/11_ protein to activate phospholipase C. Phospholipase C hydrolyzes the phospholipid phosphatidylinositol-4,5-bisphosphate into inositol 1,4,5-triphosphate (IP_3_) and diacylglycerol (DAG) [23, 25]. IP_3_, in turn, stimulates its receptors (IP_3_R) on the sarcoplasmic reticulum (SR) membrane, inducing release of stored Ca^2+^. Hence, both IP_3_ and intracellular Ca^2+^ are increased by oxytocin stimulation in a concentration-dependent fashion. Oxytocin-induced reduction of the SR Ca^2+^ reserved is thought to induce compensatory activation of store-operated calcium entry (SOCE). On the other hand, DAG-dependent phosphokinase C inhibits the activity of the Slo2.1 K^+^ channels, causing depolarization and thus activation of voltage gated calcium channels (VGCC). VGCC are essential sources of Ca^2+^ for myometrial contraction, as blocking them completely obliterates uterine contractions even in the presence of oxytocin [26]. Oxytocin can also stimulate uterine contractions by promoting prostaglandin synthesis and has been shown to induce expression of COX-2 via activation of the MAPK pathway [27]. Oxytocin receptor expression in the uterus fluctuates during the rat’s estrous cycle, with a peak during proestrus [28] right before ovulation [29]. This peak coincides with an elevation in oxytocin release. In humans, similar trends have been observed. In both rats and humans, elevation in oxytocin release has been connected to elevation in estrogen levels [30, 31]. This suggests oxytocin contributes to the regulation of fertility in rats and humans.

Given the extent of oxytocin’s impact on the regulation of uterine function, we resolved to initiate our investigation of the effect of parity on contractility by testing the hypothesis that uterine samples from non-pregnant PB rats contract more robustly in response to oxytocin than samples from non-pregnant V rats.

## Material and Methods

### Animals

All protocols and procedures were approved by the Midwestern University (MWU) Institutional Animal Care and Use Committee (IACUC; protocol # 2758). The study was carried out in accordance with the recommendation of the NIH for the care and use of Laboratory animals. Non-pregnant female CD Sprague Dawley rats were obtained from Charles River. The animals were housed in accordance with NIH guidelines for the care and use of laboratory animals, caged in pairs, and allowed food and water ad libitum. The animals were received at 16 weeks of age and kept under observation for 2 weeks, during which staging was performed daily. The first week is usually necessary for familiarizing the animals to the staging procedure that can be stressful and negatively affect their normal cycling. The rat estrous cycle is 4–5 days long. Staging by vaginal lavage was performed at the same time in the morning (~9:30) every day for 10–12 days. The myometrial tissue was harvested during the proestrus phase of the estrous cycle (identified based on the criteria described by Marcondes et al. [31] to eliminate variability in the myometrial contractility due to hormonal fluctuations). Female rats were either virgin (V) or proven breeders (PB) that experienced only one pregnancy. Age can affect the myometrial characteristics in terms of contractile protein expression as well as expression of elements of the immune system, lipid transport, and metabolism. The experimental age of 18 ± 1 weeks was chosen, because it is the youngest possible age at which female rats can give birth for the second time, according to the Charles River’s breeding program schedule. Our future research plan includes the study of the effects of parity on the performance of the pregnant uterus. Uniform ages for all study conditions will enable us to compare results more accurately.

### Tissue Dissection

Female rats in proestrus were euthanized by CO_2_ inhalation. The two uterine horns were placed in separate petri dishes containing refrigerated low-Ca^2+^-modified Krebs–Heinseleit solution (in mM, NaCl 117, KCl 4.7, NaHCO3 25, MgCl2 1.2, KH2PO4 1.2, CaCl2 0.01, glucose 11, pH = 7.4) and kept on ice. The uterine horns were then quickly dissected lengthwise along the mesometrial line. A cotton swab was then used to remove endometrial debris from the luminal aspect of the horn. One horn was weighed, flash-frozen in liquid nitrogen, and stored at −80 °C for future molecular biology studies. The other horn was cut into six longitudinal strips immediately used for contractility studies.

### Reagents

Oxytocin was obtained from Cayman Chemical Company (Ann Harbor, MI, USA). All the other reagents were from Sigma-Aldrich (St. Louis, MO, USA).

### Contractility Measurements

The uterine strips were suspended in 10 ml chambers (Myobath II, World Precision Instruments, Sarasota, FL) under a 0.5 g tension and bathed in Krebs–Heinseleit solution (in mM, NaCl 117, KCl 4.7, NaHCO3 25, MgCl2 1.2, KH2PO4 1.2, CaCl2 2.5, glucose 11, pH = 7.4, here also called regular Krebs solution) at 37 °C and bubbled with 95% O2 and 5% CO2. Force of contraction was measured with WPR force transducers connected to an IX228/S bridge amplifier (iWorx, Dover, NH; acquisition software: LabScribe, iWorx). Under these conditions, the strips contracted spontaneously and regularly. Strips that did not show adequate contractions after 15 min were considered damaged and discarded.

After an equilibration period of 30 min, the strips were challenged with 40 mM KCl to induce maximal activation. The contractility recorded under this condition was used to normalize subsequent measurements. Three subsequent 10-min washes with regular Krebs solution removed KCl and recovered normal contraction patterns. Dose response measurements followed. The six strips obtained from the uterus of each animal underwent the same treatment. Whenever preconditioning with a drug or different extracellular calcium concentration was required, strips were allowed a minimum of 30 min to adjust. A final challenge with 40 mM KCl was conducted at the end of the dose response measurements to prove sustained functional viability of the uterine strips.

LabChart 7 software (ADInstruments, Colorado Springs, Colorado) was utilized to evaluate contractility expressed as area under the curve (AUC) measured during the first 5 min after the addition of the drug. The AUC for each strip was normalized to the AUC measured for that strip during a 40 mM KCl challenge (AUC/AUC KCl).

Normalization was conducted to account for interstrip variability. Despite efforts to cut strips uniformly, post experiment measures showed that weight can vary significantly (between 0.01 and 0.08 g). Other elements of variability are the placement of suture rings to suspend the strips in the bath chambers and the alignment of the longitudinal fibers which are likely not perfectly identical. Finally, only strips that displayed regular spontaneous contractions and responded robustly to the KCl stimulation were utilized. Yet, a range in activity level from strip to strip was evident and did not strictly correlate to the weight of the strip. Normalization by the response to the KCl challenge was chosen, because it reflects the number of active and responsive myocytes in the strips.

The amplitude (g), frequency (Hz), and duration of contractile events (s) were also evaluated. Event duration was measured for spontaneous contractions and 100 nM oxytocin evoked contractions only. Duration was measured at 50% of the peak of contractions [32]. Amplitude and frequency were evaluated for all conditions.

### Statistics

Data relative to AUC/AUC in 40 mM KCl, amplitude, and frequency for the dose response to oxytocin were fitted with a sigmoid dose response curve based on the standard least square fit method:

$$y=\min +\frac{\mathit{\max}-\mathit{\min}}{1+{10}^{\left(\log EC50-x\right)}}$$

Shapiro–Wilk tests were used to analyze the normality of distribution of the data, and comparisons were conducted with a two-tailed *t*-test (statistical significance at *P* < 0.05). Differences in logEC_50_, minimal, maximal, and range response to oxytocin were evaluated and expressed as mean ± standard errors of the mean (SEM).

Data relative to contractility, amplitude, and frequency of spontaneous contractions before and after stimulus with 40 mM KCl is reported as mean ± standard deviation (Table 1). All duration data were reported as mean ± standard deviation (Table 6).

**Table 1:** Parameters describing spontaneous contractions before and after KCl challenge

Condition	Before KCl challenge				After KCl challenge			
	V		PB		V		PB	
	M	SD	M	SD	M	SD	M	SD
AUC/AUC in 40 mM KCl	0.31_a_	0.13	0.33_a_	0.12	0.27_b_	0.13	0.22_c_	0.11
AUC (g*s)	244_a_	111	240_a_	121	181_b_	99	135_c_	74
Amplitude (g)	2.78_a_	0.81	2.39_b_	0.74	2.80_a_	0.70	2.56_b_	0.71
Frequency (Hz)	0.0218_a_	0.004	0.0220_a_	0.005	0.0117_b_	0.0052	0.0103_c_	0.0072
Duration (s)	13.5_a_	2.1	14.8_a_	1.8	11.2_b_	1.7	10.6_c_	1.8

1. Obtained from 37 V (= 202 strips) and 34 PB rats (= 199 strips)

2. Durations were obtained from 500 contraction events measured in each group

3. Within each set of measures, means with different subscripts differ significantly (*P* < 0.05)

All statistical analysis was performed with Sigma Plot 14.

## Results

### Spontaneous Contractility Before and After Challenge with 40 mM KCl

The characteristics of spontaneous contractions were evaluated for all experiments with virgin (V) and proven breeder (PB) myometrial strips described in the following sections. The term “spontaneous” refers to contractions occurring in strips suspended with a passive tension of 0.5 g in Krebs solution containing 2.5 mM CaCl_2_ (regular Krebs). To assess the impact of the KCl challenge on the V and PB strips, we measured AUC/AUC in 40 mM KCl, amplitude, frequency, and duration before treatment with 40 mM KCl and after removal of the KCl solution, immediately before beginning the dose response experiments.

Table 1 reports the parameter values derived from 37 non-pregnant V rats (for a total of 200 strips) and 34 PB (194 strips) rats. Absolute AUC values are included to enable comparison with normalized data. Before the challenge with KCl, AUC/AUC in 40 mM KCl, frequency, and duration were similar in V and P, but the amplitudes recorded in V were significantly higher than in PB (<0.001). This suggests a more intense elevation in intracellular calcium in V compared to PB. Following the KCl challenge, all parameters declined significantly except amplitudes. However, V strips show stronger values than PB strips (for AUC/AUC in 40 mM KCl, *P* = 0.003; for amplitude *P* = 0.01, for frequency *P* = 0.03, and for duration *P* = 0.01). In general, the challenge with 40 mM KCl appeared to strain the myometrial strips, which did not fully recover even after prolonged (> 30 min) washes with regular Krebs solution.

### Response to Oxytocin in 2.5 mM Extracellular Calcium

**Fig. 1:**
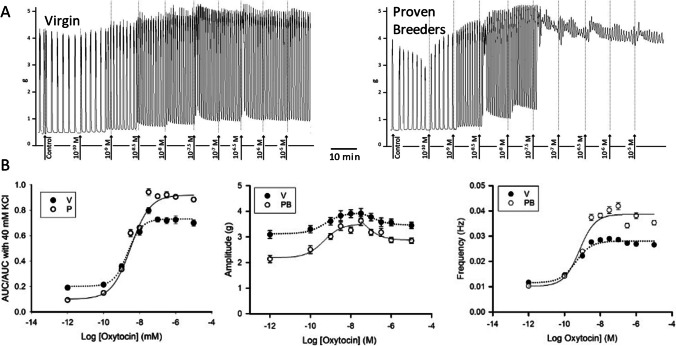
Dose response to oxytocin in 2.5 mM extracellular Ca^2+^. **A** Sample trace recorded in uterine samples from virgin (left panel) and proven breeders (right panel) female rats. **B** Dose response curves summarizing data obtained from experiment on 12 virgin (V, black circles) and 12 proven breeder (PB, white circles) rats (6 uterine strips/rat). The left panel shows the overall contractility, represented by the area under the curve over 5 min divided by the area under the curve recorded during the 40 mM KCl challenge. The middle panel shows oxytocin’s impact on contraction amplitude. The right panel represents oxytocin’s impact on contraction frequency. Error bars represent SEM. Frequency seems the most important determinant of the overall stronger contractility response to oxytocin in PB samples

Dose response to oxytocin (10^−10^ to 10^−5^ M) curves were obtained from myometrial strips from non-pregnant virgin (V) and proven breeder rats (PB) submerged in a Krebs solution containing 2.5 mM CaCl_2_ (regular Krebs). Figure 1A shows sample traces of the experiment and Fig. 1B shows the summary curves obtained under this condition (for both V and PB, rat *N* = 12). Fitting of the AUC/AUC in 40 mM KCl curves showed that oxytocin EC50 was significantly smaller in V than in PB strips (*P* < 0.001). However, both maximum and range (calculated as maximum − minimum of the curve) were more elevated in PB strips (detailed in Table 2). These variations in potency and responsiveness in the two tissue types may depend on correspondent variations in the availability and status of either the oxytocin receptors and/or of the elements of intracellular pathways activated by the binding of oxytocin to its receptors.

**Table 2:** Dose response to oxytocin parameters. Curve parameters for dose response to oxytocin in 2.5 mM Ca^2+^ (*N*_V_ = 12, *N*_PB_ = 12)

Parameter	AUC/AUC in 40 mM KCl			Amplitude (g)			Frequency (Hz)		
	V	PB	P	V	PB	P	V	PB	P
EC50	−8.68 ± 0.06*	−8.48 ± 0.06	1E^−3^	−9.2 ± 0.1	−9.3 ± 0.2	0.6	−9.3 ± 0.1	−9.2 ± 0.2	0.6
Min	0.196 ± 1E^−3^*	0.115 ± 0.02	1E^−4^	3.12 ± 0.04*	2.2 ± 0.01	5E^−14^	0.011 ± 1E^−3^	0.010 ± 3E^−3^	0.7
Max	0.756 ± 1E^−3^	0.905 ± 0.02*	5E^−5^	3.94 ± 0.03*	3.5 ± 0.1	3E^−5^	0.0281 ± 5E^−4^	0.039 ± 1E^−3^*	4E^−18^
Range	0.560 ± 1E^−3^	0.79 ± 1E^−3^*	4E^−4^	0.82 ± 0.05	1.3 ± 0.1*	2E^−5^	0.017 ± 1E^−3^	0.029 ± 3E^−3^*	8E^−5^

Throughout the dose response curve, V strips had a significantly more pronounced amplitude than PB strips (*P* < 0.001). Yet, the amplitude increase observed in PB strips in response to oxytocin was steeper than in V strips. Interestingly, the amplitude vs. [Oxytocin] curve has a hormetic trend not observed in the AUC/AUC in 40 mM KCl curve per se. After the 50 nM oxytocin dose, successive doses of the drug caused a reduction in both V and PB amplitudes.

Instead, the frequency vs. [Oxytocin] curve followed a trend very similar to that of AUC. In PB strips, maximum and range were larger than in V strip (in both cases *P* < 0.001). Durations measured for 100 nM oxytocin (see Table 6) were significantly longer in PB than in V strips (*P* = 0.002).

Hence, oxytocin induces a stronger increase of frequency and duration in PB strips, which is consistent with the AUC/AUC in 40 mM KCl trend.

Given the higher responsiveness observed in PB, we hypothesized that expression of OXTR might be more elevated after pregnancy. However, preliminary results from protein expression evaluation with ELISA did not show a significant difference. Preliminary RT-PCR measurements gave contradictory results and are currently being reevaluated. Instead, we explored the hypothesis that pregnancy induces changes in the intracellular targets of the oxytocin-activated pathway, thus causing the observed differences in the motility experiments. We speculated that variations in the availability and activation of calcium channels (Ca^2+^) and other elements regulating intracellular Ca^2+^ levels in the uterus could be different after the first pregnancy. Thus, we studied how reducing the availability of extracellular Ca^2+^, blocking intracellular Ca^2+^ channels (inositol triphosphate receptors and ryanodine receptors), and blocking the sarcoplasmic-endoplasmic reticulum Ca^2+^ ATP-ase pump (SERCA) affect the response to oxytocin in V and PB samples.

### Response to Oxytocin in 10 μM Extracellular Calcium

To test the hypothesis that the observed variations in the response to oxytocin were at least in part the result of differences in the availability of sarcolemmal voltage gated calcium channels, we repeated the dose response studies in a reduced Ca^2+^ extracellular environment. After the customary KCl challenge and subsequent recovery of rhythmic spontaneous contractions, V and PB strips were washed with an isosmotically modified Krebs solution containing 10 μM Ca^2+^. Following a minimum of 30 min of incubation, all spontaneous contractions disappeared. However, oxytocin treatment reestablished contractions, albeit to a smaller extent than with 2.5 mM Ca^2+^. In the complete absence of extracellular calcium, instead, myometrial strips did not contract either spontaneously or under uterotonic stimulation.

**Fig. 2:**
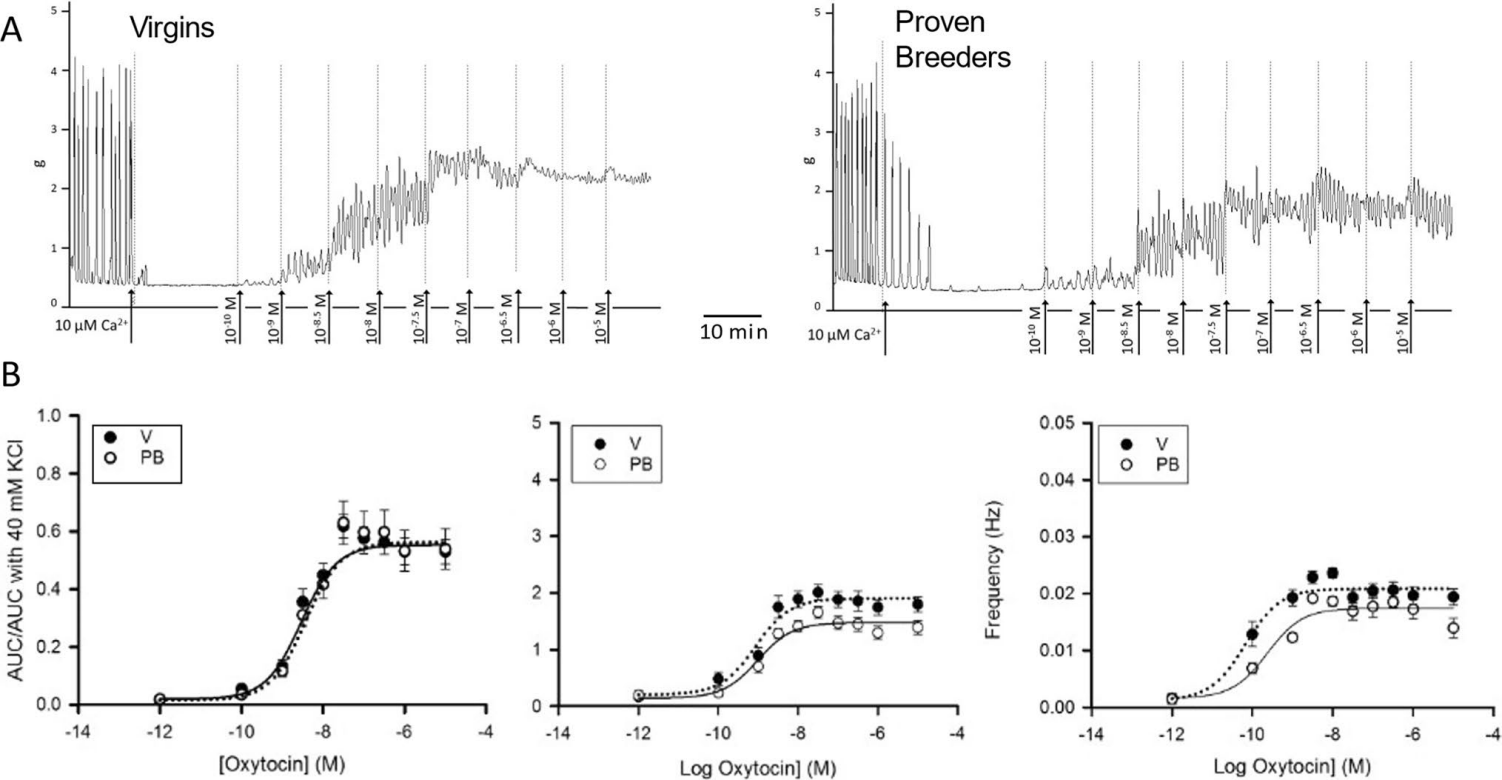
Dose response to oxytocin in 10 μM extracellular Ca^2+^. **A** Sample trace recorded in uterine samples from virgin (left panel) and proven breeders (right panel) female rats. **B** Dose response curves summarizing data obtained from experiment on 4 virgin (V, black circles) and 4 proven breeder (PB, white circles) rats (6 uterine strips/rat). The left panel shows the overall contractility, represented by the area under the curve over 5 min divided by the area under the curve recorded during the 40 mM KCl challenge. The middle panel shows oxytocin’s impact on contraction amplitude. The right panel shows oxytocin’s impact on contraction frequency. Error bars represent SEM. Reduction of extracellular Ca^2+^ eliminated differences previously observed in the dose response curves and modified the trend of the amplitude curve from hermetic to sigmoidal

As shown in Fig. 2, under these conditions, the AUC/AUC in 40 mM KCl curves were similar (for both V and PB, *N* = 4, see details on curve parameters in Table 3). Amplitude curve analysis revealed a more elevated maximal amplitude in V than in PB. However, all other curve parameters were not significantly different. Frequency curve analysis showed no significant difference between V and PB. Durations measured at 100 nM oxytocin were higher in V than in PB (*P* < 0.001), a reversal of trend compared to 2.5 mM Ca^2+^ experiments (Table 6). Compared to data from experiments conducted in 2.5 mM extracellular Ca^2+^, maximal AUC/AUC in 40 mM KCl was reduced by 19% in V and 34% in PB, maximal amplitude by 50% in V and 54% in PB, and maximal frequency by 35% in V and by 55% in PB. Instead, durations did not decline in low Ca^2+^.

**Table 3:** Dose response to oxytocin parameters. Curve parameters for dose response to oxytocin in 10 μM Ca^2+^ (*N*_V_ = 4, *N*_PB_ = 4)

Parameter	AUC/AUC in 40 mM KCl			Amplitude (g)			Frequency (Hz)		
	V	PB	P	V	PB	P	V	PB	P
Log EC50	−8.4 ± 0.1	−8.5 ± 0.1	0.5	−9.1 ± 0.1	−9.0 ± 0.2	0.7	−10.2 ± 0.3	−9.6 ± 0.4	0.6
Min	0.019 ± 0.02	0.015 ± 0.02	0.9	0.2 ± 0.1	0.1 ± 0.1	0.5	0.001 ± 2E^−3^	0.002 ± 2E^−3^	0.7
Max	0.55 ± 0.02	0.56 ± 0.02	0.7	1.90 ± 0.07*	1.48 ± 0.06	4E^−5^	0.0209 ± 6E^−4^*	0.0174 ± 8E^−4^	1E^−3^
Range	0.53 ± 0.03	0.54 ± 0.03	0.4	1.7 ± 0.1*	1.4 ± 0.1	0.04	0.019 ± 2E^−3^	0.016 ± 2E^−3^	0.1

Thus, the reduced extracellular Ca^2+^ negatively affected oxytocin-evoked contractility mainly through amplitude and frequency. Furthermore, in PB strips, the ability to respond to oxytocin was more severely compromised than in V strips. This suggests that a calcium dependent mechanism contributes to the stronger response to oxytocin reported for PB strips.

### Response to Oxytocin in 10 μM Ruthenium Red

**Fig. 3:**
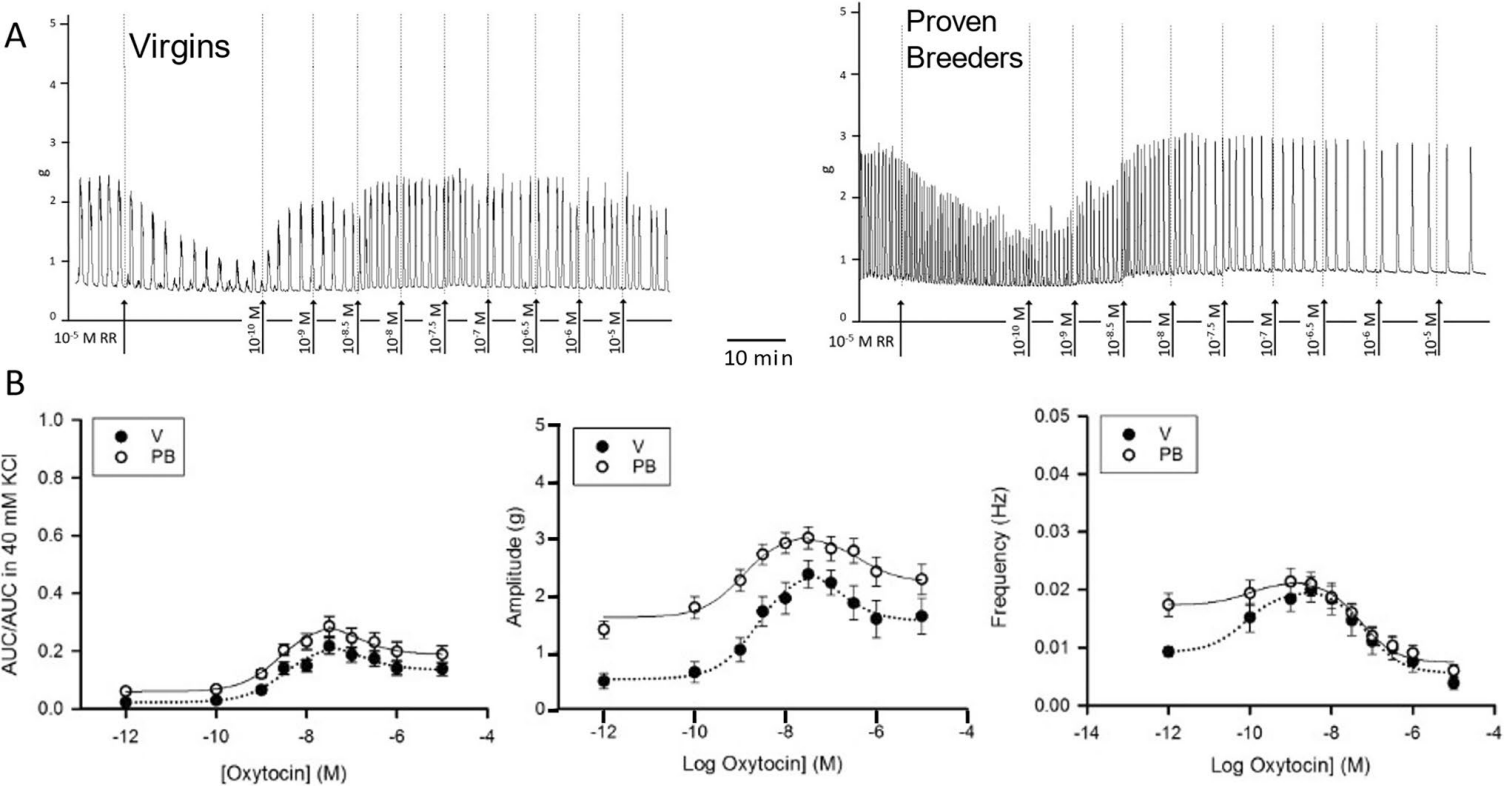
Dose response to oxytocin in 10 μM ruthenium red (RR). **A** Sample trace recorded in uterine samples from virgin (left panel) and proven breeders (right panel) female rats. **B** Dose response curves summarizing data obtained from experiment on 5 virgin (V, black circles) and 5 proven breeder (PB, white circles) rats (4–6 uterine strips/rat). The left panel shows the overall contractility, represented by the area under the curve over 5 min divided by the area under the curve recorded during the 40 mM KCl challenge. The middle panel shows oxytocin’s impact on contraction amplitude. The right panel shows oxytocin’s impact on contraction frequency. Error bars represent SEM. In the presence of high levels of RR, spontaneous activity and response to oxytocin were greatly reduced. All three dose response curves assumed a hormetic trend. The amplitude curves show that V strips were more severely affected by RR than PB strips. The frequency curves show that after a peak around 10^−7^ M oxytocin, both groups experienced a slowdown in activity that might be time dependent in nature

To test the hypothesis that Ca^2+^ release from the sarcoplasmic reticulum might be permanently altered by pregnancy, thus contributing to the observed difference in the dose response to oxytocin, the dose response study was repeated in the presence of 10 μM ruthenium red (RR). Ruthenium red is an unspecific blocker of both inositol triphosphate receptors and ryanodine receptors calcium release channels (IP_3_R and RyR). RR was added to the regular Krebs solution after strips recovered from the KCl challenge and oxytocin application were started after no less than 30 min incubation. The results of these experiments are presented in Fig. 3 and the curve parameters are detailed in Table 4. In the presence of RR, spontaneous contractions were greatly reduced, especially in V strips, yet not eliminated differently than in low Ca^2+^ experiments.

**Table 4:** Dose response to oxytocin parameters. Curve parameters for dose response to oxytocin in 10 μM ruthenium red (*N*_V_ = 5, *N*_PB_ = 5)

Parameter	Ascending branch								
	AUC/AUC in 40 mM KCl			Amplitude (g)			Frequency (Hz)		
	V	PB	P	V	PB	P	V	PB	P
Log EC50	−8.5 ± 0.2	−8.6 ± 0.1	0.3	−8.6 ± 0.1	−8.9 ± 0.2	0.06	−10.1 ± 0.2	−10.0 ± 0.2	0.8
Min	0.02 ± 0.01	0.06 ± 0.01*	4E^−3^	0.52 ± 0.08	1.61 ± 0.04*	5E^−17^	0.0092 ± 8E^−4^	0.0173 ± 5E^−4^*	2E^−11^
Max	0.22 ± 0.02	0.28 ± 0.02*	0.04	2.4 ± 0.1	3.07 ± 0.04*	2E^−8^	0.0197 ± 7E^−4^	0.0214 ± 4E^−4^*	0.03
Range	0.20 ± 0.03	0.24 ± 0.03	0.5	1.9 ± 0.1*	1.46 ± 0.06	4E^−4^	0.0105 ± 1E^−3^*	0.0041 ± 6E^−4^	7E^−7^
Parameter	Descending branch								
	AUC/AUC in 40 mM KCl			Amplitude (g)			Frequency (Hz)		
	V	PB	P	V	PB	P	V	PB	P
Log EC50	−6.8 ± 0.3	−6.9 ± 0.3	0.5	−6.8 ± 0.3	−6.5 ± 0.3	0.2	−7.1 ± 0.3	−7.3 ± 0.3	0.7
Min	0.13 ± 0.01	0.19 ± 0.01*	6E^−5^	1.6 ± 0.1	2.2 ± 0.1	0.01	0.005 ± 1E^−3^	0.0074 ± 7E^−4^	0.1
Max	0.22 ± 0.02	0.30 ± 0.01*	2E^−4^	2.5 ± 0.2	3.0 ± 0.1*	0.02	0.020 ± 1E^−3^	0.0215 ± 7E^−4^	0.3
Range	0.53 ± 0.03	0.54 ± 0.03	0.2	0.9 ± 0.1	0.8 ± 0.1*	0.6	0.015 ± 2E^−3^	0.0141± 9E^−4^	0.8

*Note:* The parameters of the dose response curves to oxytocin in ruthenium red are presented in two groups (ascending and descending branch) to account for the hormetic trend of the three curves

In addition, in RR, the PB strips displayed a significantly higher degree of spontaneous activity than V strips (*P* < 0.001). The spontaneous AUC/AUC in 40 mM KCl was reduced by 92% in V and by 62% in PB strips after addition of RR. While in 2.5 mM Ca^2+^ V had significantly higher amplitude than PB, after treatment with RR, PB displayed higher amplitude (0.035 ± 0.008 in V vs. 0.07 ± 0.008 in PB, *P* = 0.01, in both cases *N* = 5 rats = 30 strips). RR produced an 84% spontaneous amplitude reduction in V strips and only a 25% reduction in PB. The RR induced inhibition was less dramatic on spontaneous frequency, but V samples were more affected (−62%) than PB samples (−18%). Hence, PB strips displayed higher spontaneous frequency than V strips (0.009 ± 0.001 Hz in V vs. 0.017 ± 0.002 Hz in PB, *P* = 0.0006, in both cases *N* = 5 rats = 30 strips). Similarly, spontaneous duration was reduced more strongly in V (−68%) than in PB (−54%), which resulted in PB having longer durations than V strip in RR (3.6 ± 0.2 s in V vs. 4.9 ± 0.1 s in PB, *P* = 0.0001; in both cases *N* = 5 rats = 30 strips).

The maximal AUC/AUC in 40 mM KCl elicited by oxytocin were slightly yet significantly higher in PB, while the range (maximum–minimum) was similar in the two groups (see Table 4). Both maximal and range values were lower in RR than in the low calcium experiments. The overall shape of the dose response curve in RR was notably different from the dose response curves obtained in previous experiments. Curves from experiments conducted in RR were bell shaped rather than sigmoidal, and they peaked at 100 nM M oxytocin (for both V and PB; *N* = 5 rat = 30 strips).

This trend in the AUC/AUC in 40 mM curves was matched by the correspondent amplitude and frequency curves. Furthermore, in the amplitude curves, values obtained from PB strips were consistently higher than values from V strips. In the frequency curves, instead, low oxytocin doses produced a moderate increase, while the highest doses caused a definitive decline in frequency. Indeed in 10 μM oxytocin, frequencies were lower than in the absence of the drug. On the other hand, 100 nM oxytocin prolonged event durations, as expected. Yet, V strips showed longer duration than PB strips (13.6 ± 0.3 s in V vs. 11.9 ± 0.3 s in PB, *P* = 0.01; for both *N* = 5 rats = 30 strips) and in both cases, the increase was not as extensive as the one observed in 2.5 mM and 10 μM Ca^2+^.

### Response to Oxytocin in 10 μM Cyclopiazonic Acid

**Fig. 4:**
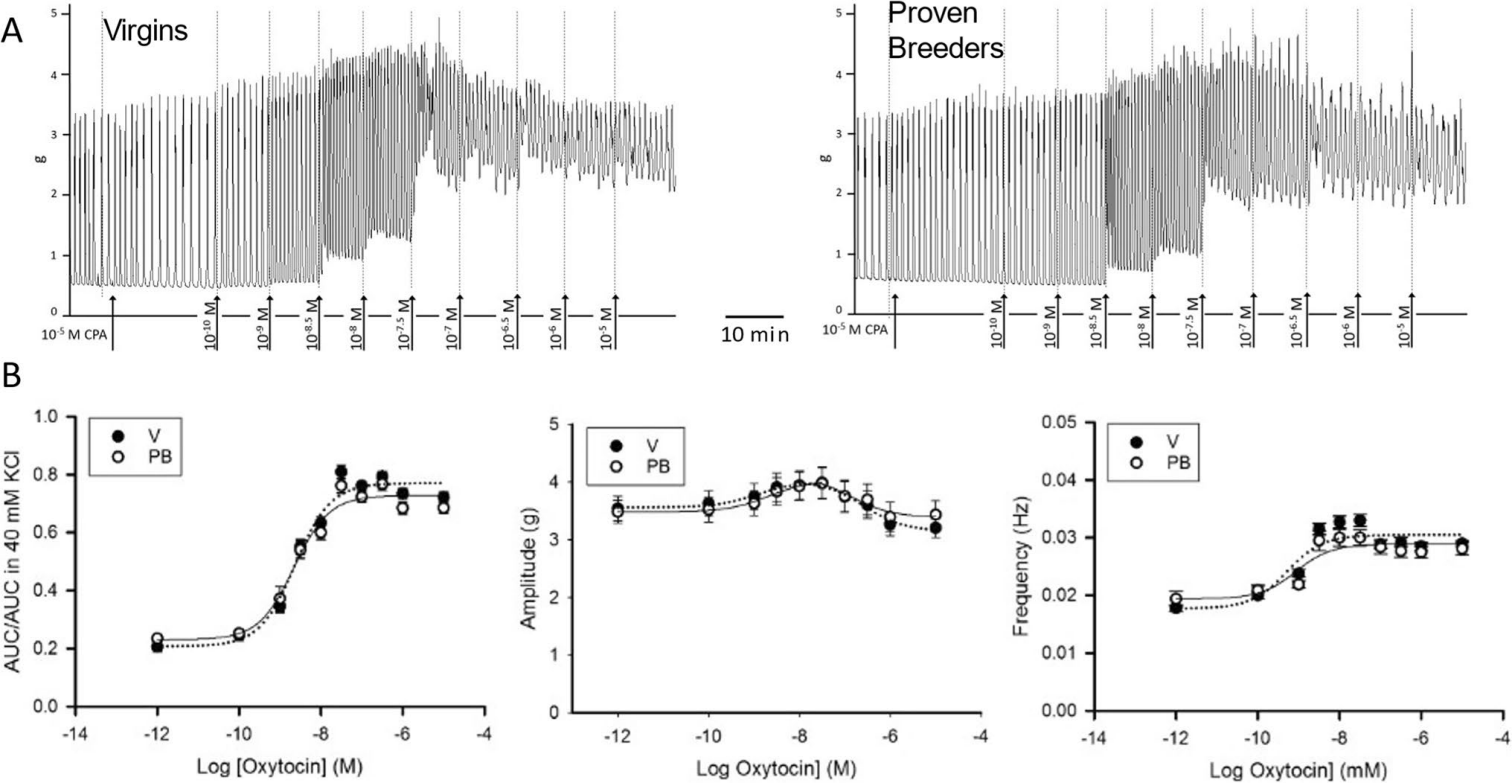
Dose response to oxytocin in 10 μM cyclopiazonic acid (CPA). **A** Sample trace recorded in uterine samples from virgin (left panel) and proven breeders (right panel) female rats. **B** Dose response curves summarizing data obtained from experiment on 4 virgin (V, black circles) and 5 proven breeder (PB, white circles) rats (6 uterine strips/rat). The left panel shows the overall contractility, represented by the area under the curve over 5 min divided by the area under the curve recorded during the 40 mM KCl challenge. The middle panel shows oxytocin’s impact on contraction amplitude. The right panel shows oxytocin’s impact on contraction frequency. Error bars represent SEM. In the presence of CPA, the general shapes of the three curves were consistent with those from Fig. 1. All differences between the two groups were eliminated, suggesting an important role for SERCA in determining the stronger response originally observed in PB samples

In addition to stimulating the activity of voltage gated calcium channels and the release of calcium from the SR, oxytocin reduces the ability of the sarcoplasmic-endoplasmic reticulum calcium ATP-ase (SERCA) to pump cytosolic calcium back into the SR. Thus, the next step was to conduct the dose response to oxytocin in the presence of a blocker of the SERCA pump, cyclopiazonic acid (CPA). Like in the case of the RR experiment, after the KCl challenge, strips were incubated for a minimum of 30 min with 10 μM CPA before treatment with cumulative doses of oxytocin. Figure 4 summarizes the findings for this experiment and the curve parameters are detailed in Table 4. The spontaneous activity of V strips recorded in the presence of CPA was similar to that observed in 2.5 mM Ca^2+^experiments. The spontaneous activity recorded in PB strips, however, was 45% more intense. This resulted in essentially identical AUC/AUC in 40 mM KCl in V and PB before addition of oxytocin (0.20 ± 0.02 in V and 0.23 ± 0.01 in PB, *P* = 0.2; *N*_V_ = 4 rats = 24 strips, *N*_PB_ = 6 rats = 35 strips). Compared to the 2.5 mM Ca^2+^ experiments, amplitude of spontaneous contractions in the presence of CPA did not change significantly in V strips. In PB strips, instead, it increased by 52%. In fact, spontaneous amplitude of V and PB was essentially identical. Frequency and event durations were similar to those registered in 2.5 mM Ca^2+^ only experiments.

All the oxytocin dose response curves obtained from V and PB strips exposed to CPA essentially overlap (see curve parameters in Table 5). This suggests a stronger dependency of PB strips on the activity of SERCA for cytosolic Ca^2+^ removal. Maximal amplitude obtained with oxytocin and spontaneous amplitude were similar in both groups. Maximal frequency was significantly more elevated in V than in PB strips (*P* < 0.001), and so was the range of activation (max–min). In 2.5 mM Ca^2+^ only experiments, the frequency trend was completely reverse. In the absence of CPA, oxytocin elicited a maximum frequency of 0.039 ± 0.001 Hz. In the presence of CPA, maximal frequency was significantly (*P* < 0.001) lower: 0.029 ± 0.001 Hz. The event durations measured at 100 nM oxytocin were longer in PB strips than in V strips (*P* < 0.001), which is well aligned with the findings in 2.5 mM Ca^2+^ only experiments (Table 6).

**Table 5:** Dose response to oxytocin parameters. Curve parameters for dose response to oxytocin in 10 μM cyclopiazonic acid (*N*_V_ = 4, *N*_PB_ = 5)

Parameter	AUC/AUC in 40 mM KCl			Amplitude (g)			Frequency (Hz)		
	V	PB	P	V	PB	P	V	PB	P
Log EC50	−8.6 ± 0.1	−8.6 ± 0.1	1	−8.9 ± 0.1	−8.7 ± 0.2	0.3	−9.3 ± 0.3	−9.1 ± 0.4	0.6
Min	0.21 ± 0.02	0.23 ± 0.02	0.1	3.6 ± 0.2	3.5 ± 0.2	0.8	0.0178 ± 5E^−4^	0.019 ± 2E^−3^	0.4
Max	0.77 ± 0.02	0.73 ± 0.02	0.1	4.0 ± 0.2	4.0 ± 0.06	0.9	0.030 ± 1E^−3^	0.029 ± 1E^−3^	0.1
Range	0.56 ± 0.04	0.50 ± 0.03	0.3	0.4 ± 0.3	0.5 ± 0.3	0.8	0.013 ± 2E^−3^	0.010 ± 1E^−3^	0.1

**Table 6:** Durations

Condition	No oxytocin						100 nM oxytocin					
	V			PB			V			PB		
	M (s)	SD	*N*	M (s)	SD	*N*	M (s)	SD	*N*	M (s)	SD	*N*
2.5 mM Ca^2+^	11.2_a_	1.7	500	10.6_b_	1.8	500	16.4_c_	5.2	348	18.1_d_	4.8	350
10 μM Ca^2+^	N/A	N/A	2	N/A	N/A	1	20.1_c_	3.8	155	16.9_d_	4.2	100
10 μM RR	3.6_a_	1.8	66	4.2_b_	2.2	71	13.6_c_	3.0	100	11.8_d_	4.2	110
10 μM CPA	10.8_a_	3.0	232	11.2_a_	3.0	235	15.0_b_	7.0	115	21.0_c_	3.1	116

1. *N* = number of contraction events measured

2. 2.5 mM Ca^2+^ = spontaneous contraction after KCl challenge (37 V rats; 34 PB rats)

3. 10 μM Ca^2+^ = (4 V rats; 4 PB rats) almost no contractions were recorded in the absence of oxytocin

*4. RR*, ruthenium red (5 V rats; 5 PB rats)

*5. CPA*, cyclopiazonic acid (4 V rats; 5 PB rats)

6. Within each set of measures, means with different subscripts differ significantly (*P* < 0.05)

## Discussion

This work tested the hypothesis that pregnancy induces epigenetic changes in the uterus that permanently modify its contractility properties. A variety of experimental approaches can be adopted to test this idea. We chose to study the motility response of uterine samples from non-pregnant virgin (V) and proven breeder (PB) female rats to oxytocin, since this hormone is both a major regulator of uterine contractility and a uterotonic agent commonly utilized in clinical practice. We found that, although the uterine affinity for oxytocin was not modified by pregnancy, the responsiveness to oxytocin was stronger in PB tissues. This suggested that the experience of pregnancy produces a permanent elevation in the number of available oxytocin receptors and/or elements of the intracellular second messenger-activated pathways triggered by oxytocin. The present paper focused on this latter aspect. In summary, we found evidence that PB uteri might be richer in voltage gated Ca^2+^ channels and in sarcoplasmic endoplasmic reticulum Ca^2+^ ATPase (and/or possibly in the plasma membrane Ca^2+^ ATPase and sodium calcium exchanger), and poorer in SR release channels than V uteri.

The role of extracellular Ca^2+^ entry as an essential drive of myometrial contractility is well established [33]. Calcium entry is mediated by VGCC and SOCE [34]. Among the VGCC, both L-type calcium channels and T-type calcium channels contribute to Ca^2+^ entry [35]. We explored the general impact of extracellular Ca^2+^ availability on myometrial contractility of V and PB tissues. Complete elimination of extracellular Ca^2+^ abolished any spontaneous or oxytocin-induced contraction. This was an expected result, as the pivotal role of extracellular Ca^2+^ in myometrial contractility is well established. In as little as 10 μM extracellular Ca^2+^, uterine samples displayed the ability to respond to oxytocin, but they did not spontaneously contract. The lack of spontaneous contractions suggests that 10 μM extracellular Ca^2+^ do not sustain an adequate Ca^2+^ influx. Hence, the main mechanism driving contraction in the presence of oxytocin seems to be Ca^2+^ release from the SR. The dose response to oxytocin showed a reduction in the amplitude and frequency in both sample types. However, this reduction was more severe in PB strips. Based on this, we hypothesize that PB strips release SR Ca^2+^ less efficiently because of lower expression/activation of inositol-3-phospate receptor channels (IP_3_R) or lower expression/activation of ryanodine receptor channels (RyR). In 2.5 mM extracellular Ca^2+^, this difference is obscured by the strong inward Ca^2+^ currents through VGCC.

Treatment of the myometrial preparations with 10 μM ruthenium red (RR) induced a robust decrease in AUC, with a stronger depression in amplitude than in frequency and duration. RR is a classical inhibitor of RyRs; however, it has been shown to affect at micromolar concentrations other cellular structures, notably IP_3_R channels [36, 37] and VGCCs [37]. Intracellular RR concentrations >20 μM may result in the partial blockage of BK channels [37], the membrane Ca^2+^ pump [38], Ca^2+^ uptake in the mitochondria [39], the formation of the Ca^2+^-calmodulin complex [40], and myosin light chain phosphatase [41]. In the experiments presented here, we can confidently state that the effects were mostly confined to the inhibition of SR channels.

Spontaneous activity was stronger than in 10 μM Ca^2+^, albeit much reduced compared to the experiments conducted in 2.5 mM Ca^2+^ only. This indicates a substantial reduction of the available intracellular Ca^2+^. Yet, there was enough Ca^2+^ influx to support contraction. We cannot completely exclude the possibility that some inhibition of VGCCs occurred; however, the most prevalently affected mechanisms here were Ca^2+^ release via RyRs and IP_3_R activation. If RR had blocked VGCCs, no spontaneous activity would have been recorded, as seen in the low Ca^2+^ experiments. The fact that V samples displayed lower amplitude and frequency than PB supports the idea that V uteri might have a higher expression of SR channels than PB uteri. Thus, RR affects more V than PB samples.

Exposure of RR-treated strips to oxytocin elicited a weaker response than non-pretreated samples, regardless of extracellular Ca^2+^ availability. This suggests that the mechanisms inhibited by RR play a major role in the action of oxytocin on the rat myometrium, in accordance with published literature [42].

A peculiarity of these curves is that they are not sigmoidal. An initial increase in activity is followed by a deflection at higher oxytocin concentrations. In control experiments, the amplitude curve showed this hormetic trend, which we attributed to a partial progressive depletion of the SR under the persistent oxytocin stimulation. In those experiments, though, sustained frequencies compensated for the hermetic trend of the amplitude and the overall AUC/AUC in 40 mM KCl curves were sigmoidal. Instead, in the presence of RR, also frequencies abate significantly at higher oxytocin doses. Furthermore, this decline is identical in V and PB.

We first hypothesized that this phenomenon could be time dependent. We speculated that the polycationic nature of RR [43] would cause its intracellular concentrations to rise slowly, thus delaying some effects of the drug. To test this, we repeated the experiments with RR initiating the oxytocin dose response curve after a 2-h interval (data not shown). No significant difference was detected in any of the parameters for all curves and the non-sigmoidal trend in AUC, amplitude, and frequency was maintained. Therefore, a different mechanism must be involved. For example, the absence of adequate SR Ca^2+^ release could trigger a downregulation of oxytocin receptors. Further investigation with more specific inhibitors of RyRs (e.g., ryanodine) and IP_3_R (e.g., xestospongin) might help explain this finding.

In the last set of experiments, we investigated the hypothesis that the sarcoplasmic-endoplasmic reticulum Ca^2+^ ATP-ase (SERCA) contributes to the different responses to oxytocin observed in V and PB samples. Together with the plasma membrane Ca^2+^-ATPase (PMCA) and the sodium calcium exchanger (NCX), SERCA contributes to intracellular Ca^2+^ decay and relaxation in the myometrium [44, 45]. Furthermore, SERCA participates in the regulation of SOCE. SOCE is activation by depletion of intracellular calcium stores. This depletion can be mediated by opening of IP_3_R and RyR channels, Ca^2+^ leak, and SERCA inhibition [46]. Oxytocin is known to stimulate SOCE through TRPC proteins [47], it can also do so indirectly via IP_3_R activation [35]. No direct effect of oxytocin on SERCA or its physiological modulators (e.g., phospholamban) has been documented so far. Wray and Burdyga [44] argued that the vast conformational changes the pump undergoes as it transports ions across the SR membrane limit the extent of physiological modulations that can affect it. However, manipulation of SERCA may offer an insight in possible alterations of Ca^2+^ homeostasis occurring in the myometrium in the presence of oxytocin.

Application of 10 μM CPA, a reversible SERCA blocker, increased the spontaneous activity of the myometrial samples compared to the control experiments (i.e., no CPA, 2.5 mM Ca^2+^). Amplitude (which reflects cytosolic Ca^2+^ levels) was the parameter most affected. This is consistent with the decrease efficiency of Ca^2+^ removal caused by blocking SERCA. This change was significant in PB, not in V strips. In control experiments, the spontaneous amplitude recorded in PB strips was significantly lower than in V strips. The strong increase in PB amplitude observed with CPA could signify that SERCA is more expressed and/or more active in these samples. Without CPA, removal of cytosolic Ca^2+^ and replenishment of the SR might be quicker, which could also lead to a reduction in the activation of SOCE and an overall reduced availability of cytosolic Ca^2+^.

An alternative explanation involves the coordinated activity of SERCA, PMCA, and NCX. It has been shown that large peripheral portions of the myometrial SR are in close proximity to caveolae-rich regions of the plasma membrane, where PMCA and NCX cluster [44, 45]. It has been proposed that after having been quickly sequestered by SERCA, Ca^2+^ is quickly released from the SR at these caveolae-rich sites. PMCA and NXC would then immediately extrude it from the cell [44]. Another hypothesis to explain the CPA data is that, in PB uteri, the expression of PMCA and/or NCX is enhanced. By blocking SERCA, we might have indirectly impaired the function of these structures. Oxytocin is known to inhibit PMCA; thus, this alternative possibility is worth considering.

AUC/AUC in 40 mM Ca^2+^, amplitude, frequency, and duration recorded during the dose response to oxytocin were very similar in both groups. In V strips, the dose response to oxytocin curve was substantially unchanged by CPA. In PB strips, oxytocin application did not induce the dramatic increase in frequency and AUC/AUC in 40 mM KCl previously observed in control experiments.

If SERCA is more expressed or more functional in the PB uterus, it could limit the amplitude of spontaneous contractions. Increasing levels of oxytocin would release this inhibition. Compounding this effect with the activation of VGCC (which we earlier hypothesized might also be more expressed or activated in PB) could explain the stronger response to oxytocin observed in PB strips.

The oxytocin effects on SERCA and its physiological modulator phospholamban in general are not considered significant in the myometrium [44]. Yet, oxytocin could exert more influence on the activity of SERCA than currently thought. This idea is supported by a 1991 paper by Magocsi et al. [48] that showed a 34% reduction Ca^2+^ uptake in SR vesicles pretreated with oxytocin. It has been shown that 17beta-estradiol upregulates SERCA2a in cultured cells and that in pregnancy and labor, the expression of SERCA in the rat myometrium progressively increases [49]. It is possible that the prolonged estradiol stimulation during gestation might permanently affect the expression of the pump. Also, the expression and activities of other modulators of the pump might be modified by pregnancy. Based on these considerations, investigation of the expression levels of SERCA and its modulators in the V and PB is important to clarify this mechanism. Again, this does not exclude the possibility that PMCA and NCX are also expressed differently in V and PB uteri, thus we intend to explore it in parallel in future studies.

Another consideration is that CPA induces depletion of the SR without affecting the PMCA, the Na^+^/Ca^2+^-ATPase, the H^+^/K^+^-ATPase, or the mitochondrial F1-ATPase [50]. Therefore, here CPA removed the contribution of the SR to spontaneous and oxytocin evoked contractions. Consequently, alterations of intracellular Ca^2+^ management and specifically elements of the SR could be the main determinants of the different response to oxytocin in V and PB strips.

In conclusion, this paper presents evidence that pregnancy induces permanent alterations to the uterine tissue. Our focus was on the contractility properties of the myometrium and its response to oxytocin. We presented motility data indicating that the most significant changes in this area concern mechanisms of Ca^2+^ handling, especially at the SR level. These alterations are likely at the base of clinical observations. For example, the more powerful response to oxytocin observed in PB tissue at least in part explains the more intense postpartum lower abdominal pains experienced by parous mothers. These pains are important for the involution process and are caused by powerful uterine contractions associated with oxytocin release, especially during lactation [8, 9]. The decrease incidence of dysmenorrhea is likely related to a different post pregnancy ability to produce prostaglandins that has not been explored in this study. Finally, the risk for uterine atony has been shown to be increased by parity. This potentially lethal condition is typically associated with prolonged or precipitous labor, uterine distension, and extended stimulation with exogenous oxytocin [51, 52]. This suggests that fatigue or an oxytocin receptor downregulation process might be involved. An enhanced response to oxytocin in parous myometrium might indeed favor and accelerate this type of process.

Undoubtably, more work, especially in the molecular biology area, is required to tease out the specific structures and processes affected, based on the hypotheses we formulated in this discussion. Continued efforts in this line of research promise an advancement of our understanding of both the impacts of pregnancy on the function and health of the uterus and, more generally, of the regulation of myometrial contractility.

## Data Availability

Not applicable.

## Declarations

### Animal Research

IACUC Protocol #2758 with IACUC committee approval included.
